# Comparison of high‐power short‐duration and low‐power long‐duration radiofrequency ablation for treating atrial fibrillation: Systematic review and meta‐analysis

**DOI:** 10.1002/clc.23493

**Published:** 2020-10-27

**Authors:** Chao‐feng Chen, Jing Wu, Chao‐lun Jin, Mei‐jun Liu, Yi‐zhou Xu

**Affiliations:** ^1^ Department of Cardiology Hangzhou First People's Hospital Hangzhou Zhejiang China; ^2^ Department of Cardiology Nanjing Medical University Nanjing Jiangshu China

**Keywords:** atrial fibrillation, efficacy, high‐power short‐duration ablation, low‐power long‐duration ablation, safety

## Abstract

**Background:**

High power shorter duration (HPSD) ablation seen to increase efficacy and safety treating of atrial fibrillation (AF); however, comparative data between HPSD and low power longer duration (LPLD) ablation are limited.

**Hypothesis:**

We thought that HPSD might bring more clinical benefits. The aim of this meta‐analysis was to evaluate the clinical benefits of HPSD in patients with AF.

**Methods:**

The Medline, PubMed, Embase, and the Cochrane Library databases were searched for studies comparing HPSD and LPLD ablation.

**Results:**

Ten trials with 2467 patients were included in the analysis. Pooled analyses demonstrated that HPSD showed a benefit of first‐pass pulmonary vein isolation (PVI) (risk ratio [RR]: 1.20; 95% confidence interval [CI]: 1.10‐1.31, *P* < .001) and recurrence of atrial arrhythmias (RR: 0.73; 95% CI: 0.58‐0.91, *P* = .005). Additionally, HPSD could reduce procedural time (weighted mean difference [WMD]: −42.93; 95% CI, −58.10 to −27.75, *P* < .001), ablation time (WMD: −21.01; 95% CI: −24.55 to −17.47, *P* < .001), and fluoroscopy time (WMD: −4.11; 95% CI: −6.78 to −1.45, *P* < .001). Moreover, major complications and esophageal thermal injury (ETI) were similar between two groups (RR: 0.75; 95% CI: 0.44‐1.30, *P* = .31) and (RR: 0.57; 95% CI: 0.21‐1.51, *P* = .26).

**Conclusions:**

HPSD was safe and efficient for treating AF. Compared with LPLD, HPSD was associated with advantages of procedural features, higher first‐pass PVI and reducing recurrence of atrial arrhythmias. Moreover, major complications and ETI were similar between two groups.

## 1 INTRODUCTION

Radiofrequency catheter ablation (RFCA) is an effective treatment for atrial fibrillation (AF).^1^ The efficacy of RFCA is associated with transmural, continuous, and permanent cellular damage.^2^ Despite the technical advancement in force sensing and stability monitoring, the rate of pulmonary vein (PV) reconnection remains frequently by low‐power longer‐duration (LPLD) ablation.

Thermal injury by RFCA involves two consecutive phases: resistive and conductive. The balance between the power and duration parameters in resistive and conductive heating has a significant influence on lesion creation. Resistive heating immediately causes irreversible myocardial tissue injuries with cellular death, whereas conductive heating passively extends to deeper tissue layers, causing potential reversible tissue damage. LPLD is associated with longer conduction heating, and the heating of the deeper tissues increases with longer durations of radiofrequency (RF) application.^2^, ^3^, ^4^, ^5^, ^6^ The left atrium is adjacent to the esophagus and the injury depth may be excessive from long‐duration ablation, both of which increase the risk of esophageal thermal injuries (ETIs).^7^ The incidence of atrio‐esophageal fistula (AEF) was reported as 0.1% to 0.25%, and the incidence of ETI was 2% to 50% for the LPLD^8^.

To optimize AF ablation, a novel energy delivery strategy with high‐power shorter‐duration (HPSD) ablation was used for AF treatment.^3^ Some studies found that HPSD ablation could increase efficacy and minimize deep tissue injuries. However, the data comparing HPSD and LPLD were limited and inconsistent. Therefore, this meta‐analysis was performed to evaluate the efficacy and safety of HPSD compared to LPLD in treating AF.

## 2 METHODS

### 2.1 Data sources and search strategy

Relevant studies were sourced from Medline, PubMed, Embase, the Cochrane Library, and Elsevier's ScienceDirect databases. Reports published in nonEnglish languages were excluded from the search. The search strategy employed relevant keywords and medical subject heading (MeSH) terms, including the following: ([Atrial fibrillation] OR [AF]) and ([Radiofrequency] OR [RF] OR [Catheter ablation]) and ([High power] OR [High‐power shorter‐duration] OR [HPSD]) and ([Low power] OR [low‐power long‐duration] OR [LPLD] OR [conventional power]). The literature search was updated in September 2020.

### 2.2 Inclusion and exclusion criteria

Two reviewers (Chao‐feng Chen and Jing Wu) screened and identified studies that met the following inclusion criteria: (a) patients with drug‐refractory symptomatic AF who underwent RF ablation; (b) wide‐area circumferential ablation for pulmonary vein isolation (PVI) applied using irrigated‐tip catheters; (c) patients undergoing treatment using catheter ablation (CA) for the first time; (d) comparison between HPSD and LPLD; (e) sample size ≥20; and (f) studies needed to provide at least one reliable piece of information regarding procedural outcomes, complications, ETI, first‐pass PVI, and follow‐up in both groups. The exclusion criteria were as follows: (a) an equivocal study design or group allocation, and (b) conference abstracts, case reports, case series studies, editorials, review articles, or nonEnglish language articles.

### 2.3 Quality assessment and data extraction

The study quality was evaluated by two investigators (Mei‐jun Liu and Chao‐lun Jin) using the Newcastle‐Ottawa Scale (NOS) for observational studies and the Delphi consensus criteria for randomized controlled studies (RCTs). The NOS system consisted of eight questions with nine possible points. A star system was used to judge the data according to the selected populations, comparability of the groups, and exposure/outcome of interest. A study with NOS ≥7 was judged to be a study of good quality.^9^ The Preferred Reporting Items for Systematic Reviews and Meta‐analyses Amendment to the Quality of Reporting of Meta‐analyses Statement and recommendations from the Cochrane Collaboration in epidemiology were followed. Data extraction was conducted by mutual agreement, and all potential disagreements were resolved by consensus.^10^, ^11^

### 2.4 Definitions

HPSD: Ablation power > 40 W, with ablation duration of 2 to 10sper site.

LPLD: Ablation power limited to 20‐40 W, with a longer ablation duration of 10 to 30s per site.

Procedure time: Time from the application of local anesthesia to the withdrawal of all catheters.

Ablation time: Time from the first to the last application.

Fluoroscopy time: Time for fluoroscopy from the start to the end of the procedure.

Atrial arrhythmias recrudescence: Any symptomatic or asymptomatic atrial arrhythmia lasting >30s after completing the blanking period post CA.

First‐pass PVI: Rate of complete PVI after first‐pass circumferential RF delivery.

Major complications: Complications that required any intervention or prolonged hospital stay.

ETI: Endoscopy or MRI late gadolinium enhancement (LGE) were performed to assess esophageal thermal injury post CA. ETI was categorized as mild (LEG MRI shows minimal or focal LGE; endoscopy shows hematoma or minimal erosion [<3 mm]), moderate (LEG MRI shows transmural or nearly transmural LGE of the anterior wall; endoscopy shows erosion), and severe (LEG MRI shows transmural LGE involving more than 5 mm of tissue or in more than one location; endoscopy shows an ulcer).

### 2.5 Assessment of heterogeneity reported bias and statistical analysis

All statistical analyses were performed using RevMan version 5.3 (Nordic Cochrane Center; The Cochrane Collaboration, 2014). The statistical analysis was completed by an independent statistician (Chao‐feng Chen). Odds ratios (ORs) or risk ratios (RRs) with 95% confidence intervals (CIs) were used as risk estimates and were pooled by the software. Continuous variables were analyzed using weighted mean differences (WMD). Random‐effect or fixed‐effect models used weightings based on an inverse variance, which were calculated according to the method by DerSimonian and Laird.^12^ The heterogeneity of studies was evaluated by Cochran's Q and the I^2^ statistic. An I^2^ value >50% was defined as significant heterogeneity. If there have significant heterogeneity, the random‐effects model was used.^13^

## 3 RESULTS

### 3.1 Eligible studies

**FIGURE 1:**
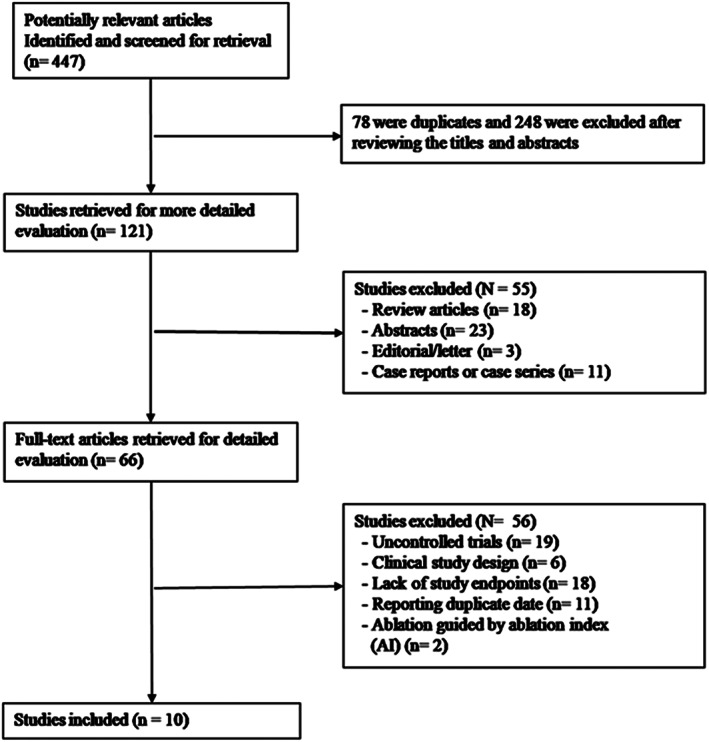
Flow diagram of study selection process

A flowchart of the detailed search process is illustrated in Figure 1. Initially, 447 potentially studies were identified, of which 78 were duplicates and 248 were excluded after reviewing the titles and abstracts. Of the remaining 121 studies, 18 review articles, 3 editorial/letters, 11 case reports or case series stidies, and 23 abstracts were excluded. Next, 56 studies were excluded after a detailed evaluation of the full text for the following reasons: 19 were uncontrolled studies, 6 were clinical studies design, 18 lacked study endpoints, and 11 reported duplicate data. And then, two trails by Berte B et al^14^ and Dhillon G et al^15^ comparing HPSD and LPLD were excluded because their ablations were guided by the ablation index (AI) without illustrating ablation time per site details. Consequently, 10 clinical trials with 2467 patients were enrolled in this meta‐analysis.^8^, ^16^, ^17^, ^18^, ^19^, ^20^, ^21^, ^22^, ^23^, ^24^

### 3.2 Study characteristics

The characteristics of the included trials and ablation settings are shown in Table 1 and Table S1 (Supplementary file). A total of 2467 patients were enrolled in these trials (1466 in the HPSD group and 1001 in the LPLD group). The mean ages of the study participants ranged from 58.7 ± 11.1 to 69 ± 11.8 years, and the mean follow‐up duration was from 10 months to 36 months. Some trails included other characteristics like as left atrial size, left atrial volume, rate of hypertension, diabetes mellitus, coronary artery disease, heart failure and transient ischemic attack/stroke. Six of ten were prospective studies^17^, ^18^, ^19^, ^20^, ^21^, ^23^, and one was RCT.^21^ The meta‐regression analysis revealed that no baseline characteristics could affect the correlation between two groups. A visual inspection of the funnel plot including all studies showed symmetry, indicating a low risk of publication bias (Appendix S1 Supplementary file). Moreover, in the most of studies consecutive patients receiving HPSD were compared with patients who were age and sex matched but treated with LPLD. Additionally, all patients received PVI, and additional linear ablations were performed in select patients at the operator's discretion in some trials.^8^, ^16^, ^18^, ^21^, ^24^ All studies had good methodological qualities. The grouping results ensured the feasibility of this meta‐analysis.

**TABLE 1:** Baseline characteristics of included study

Trial (year)	Country	Treatment group	Patients (n)	Age (y)	Male (n, %)	Paroxysmal (n, %)	LA size (mm)	LVEF (%)	Hy (n, %)	DM (n, %)	CAD (n, %)	HF (n, %)	Stroke/TIA (n, %)	Follow	Design	NOS
Baher 2018	United States, Germany	HPSD	574	69 ± 11.8	385 (67.1)	276 (46.8)	NR	NR	369 (64.2)	112 (19.5)	130 (22.6)	89 (15.5)	81(14.1)	2.5Y	Retrospective	8
		LPLD	113	68.3 ± 11.6	67 (59.3)	80 (70.8)	NR	NR	68 (60.1)	18 (18.5)	20 (17.7)	15 (13.2)	7 (6.2)			
Bunch 2019	United States	HPSD	402	67.1 ± 10.5	253 (62.9)	190 (47.3)	NR	54.6 ± 12.1	358 (89.1)	126 (31.3)	54(13.5)	190(47.3)	47(11.7)	3Y	Retrospective	7
		LPLD	402	66.4 ± 12.2	262 (65.2)	202 (50.2)	NR	54.7 ± 12.8	348 (86.6)	121(30.1)	50(12.4)	188(46.8)	51(12.7)			
Castrejón‐Castrejón 2020	Spain	HPSD	48	61 ± 10	32 (67.0)	31 (65.0)	NR	57 ± 9	NR	NR	NR	NR	NR	NR	Prospective, non‐randomized	8
		LPLD	47	60 ± 10	28 (60.0)	30 (64.0)	NR	56 ± 11	NR	NR	NR	NR	NR			
Kottmaier 2020	Germany	HPSD	97	60.8 ± 13.9	57 (58.8)	97 (100.0)	NR	57 ± 5	56(57.7)	NR	13(13.4)	NR	6(6.2)	12 M	Prospective, non‐randomized	9
		LPLD	100	60.8 ± 10.5	60 (60.0)	100 (100.0)	NR	55 ± 9	58(58)	NR	9(9)	NR	7(7.0)			
Pambrun 2019	France	HPSD	50	65 ± 8.2	35 (70.0)	50 (100.0)	107.6 ± 23.1^a^	61.7 ± 5.6	14(28)	3(6)	2 (4)	NR	3(6%)	12 M	Prospective, non‐randomized	9
		LPLD	50	62.5 ± 10.6	30 (60.0)	50 (100.0)	102.9 ± 20.1^a^	61.1 ± 4.4	12(24)	3(6)	0 (0)	NR	3(6%)			
Vassallo 2019	Brazil	HPSD	41	64 ± 10	34 (83.0)	28 (68.3)	43.3 (28‐62)	NR	33(80)	18(43.9)	NR	NR	3(7.3%)	12 M	Retrospective	8
		LPLD	35	61 ± 12	22 (64.7)	27 (77.0)	41.9 (23‐56)	NR	26(64.3)	8(22.8)	NR	NR	3(8.6%)			
Yazaki 2020	Japan	HPSD	32	61 ± 11	27 (84.0)	22 (89.0)	40 ± 13	55 ± 7	NR	NR	NR	NR	NR	10 M	Retrospective	8
		LPLD	32	66 ± 11	20 (63.0)	29 (91.0)	41 ± 14	56 ± 7	NR	NR	NR	NR	NR			
Shin DG 2020	Korea	HPSD	50	58.5 ± 7.9	39 (78.0)	25 (50.0)	39.9 ± 4.6	55.7 ± 11.4	24(48)	8(16)	NR	13(26)	7(14)	12 M	RCT	9
		LPLD	50	58.7 ± 11.1	33 (66.0)	24 (48.0)	40.7 ± 6.5	58.9 ± 8.3	22 (44.0)	8 (16.0)	NR	5 (10.0)	6 (12)			
Ejima K 2020	Japan	HPSD	60	63.0 ± 11.3	44 (73.0)	60 (100.0)	34.3 ± 10.3*	57.7 ± 3.9	29 (48)	10 (17)	NR	NR	6 (10)	20.7 ± 2.0 M	prospective cohort study,	9
		LPLD	60	66.7 ± 8.9	42 (70.0)	60 (100.0)	36.1 ± 8.7*	57.4 ± 6.3	30 (50)	12 (20)	NR	NR	7 (12)			
Yavin H 2020	United States	HPSD	112	62.3 5.2	76 (67.8)	76 (67.9)	44.2 4.7	60.3 6.1	70 (62.5)	11 (9.8)	NR	NR	NR	1.2(0.16 ~ 2.92)Y	Prospective non‐randomized	9
		LPLD	112	64.8 7.2	67 (59.8)	67 (59.9)	47.1 5.1	57.8 5.4	76 (67.8)	7 (6.2)	NR	NR	NR	1.9(0.25 ~ 3.66)Y		

*Note*: Values are reported as the mean ± SD, medians (interquartile range), or n (%).

Abbreviations: CAD, coronary artery disease; CRT, prospective randomized controlled trial; DM, Diabetes mellitus; HF, heart failure; HPSD, High power shorter duration; Hy, hypertension; LA, left atrium; LPLD, low power longer duration; M, months; NOS, Newcastle‐Ottawa Quality Assessment Scale. NR, not recorded; TIA, transient ischemic attack; Y, years.

^a^ Evaluate left atrial by left atrial volume.

### 3.3 Clinical outcomes

**FIGURE 2:**
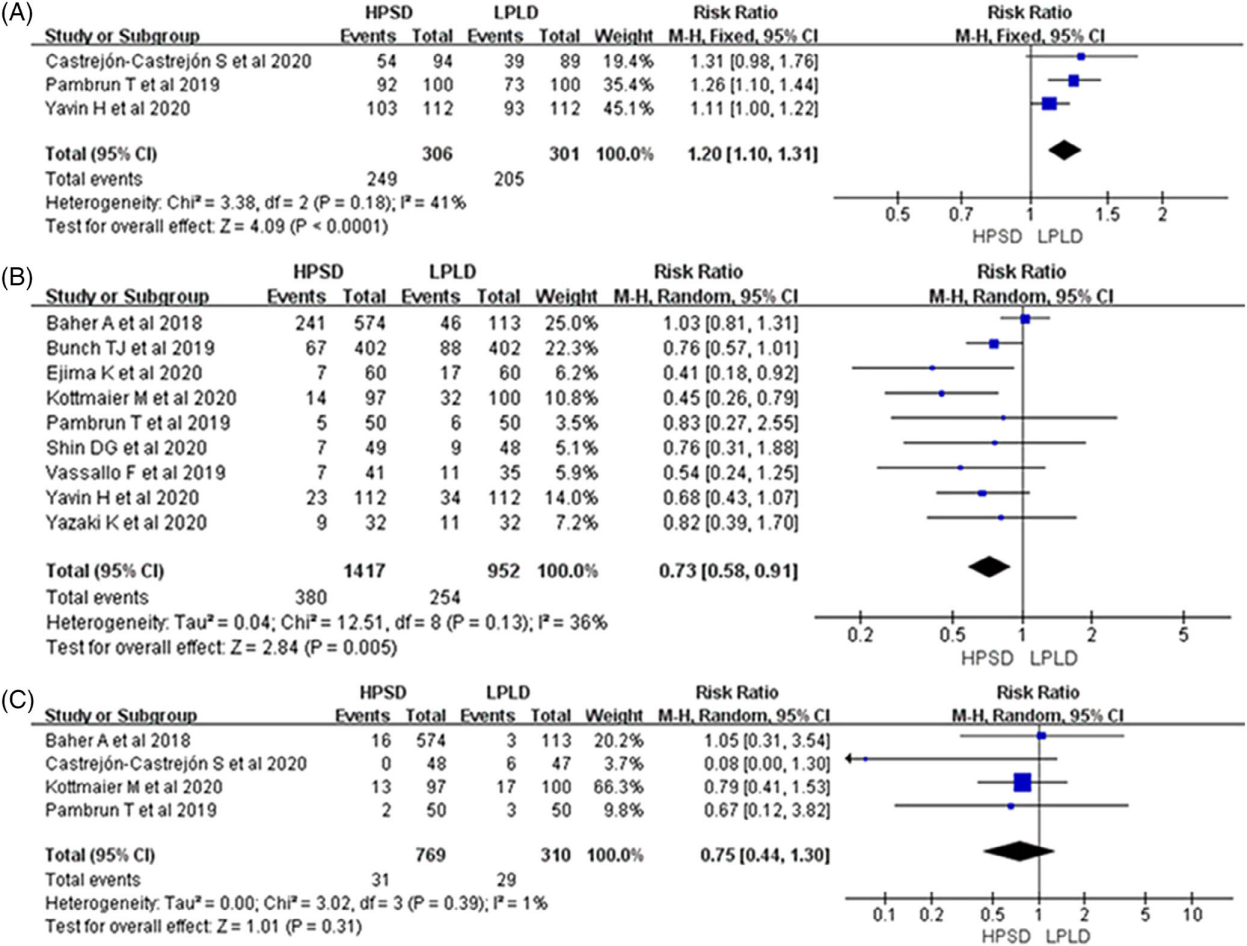
Forest plots of first‐pass PVI of PVs, A; recurrence of atrial arrhythmias, B; and major complications, C, for HPSD vs LPLD. HPSD, high power shorter duration; LPLD, low power longer duration; PVI, pulmonary vein isolation; PVs, pulmonary veins

**FIGURE 3:**
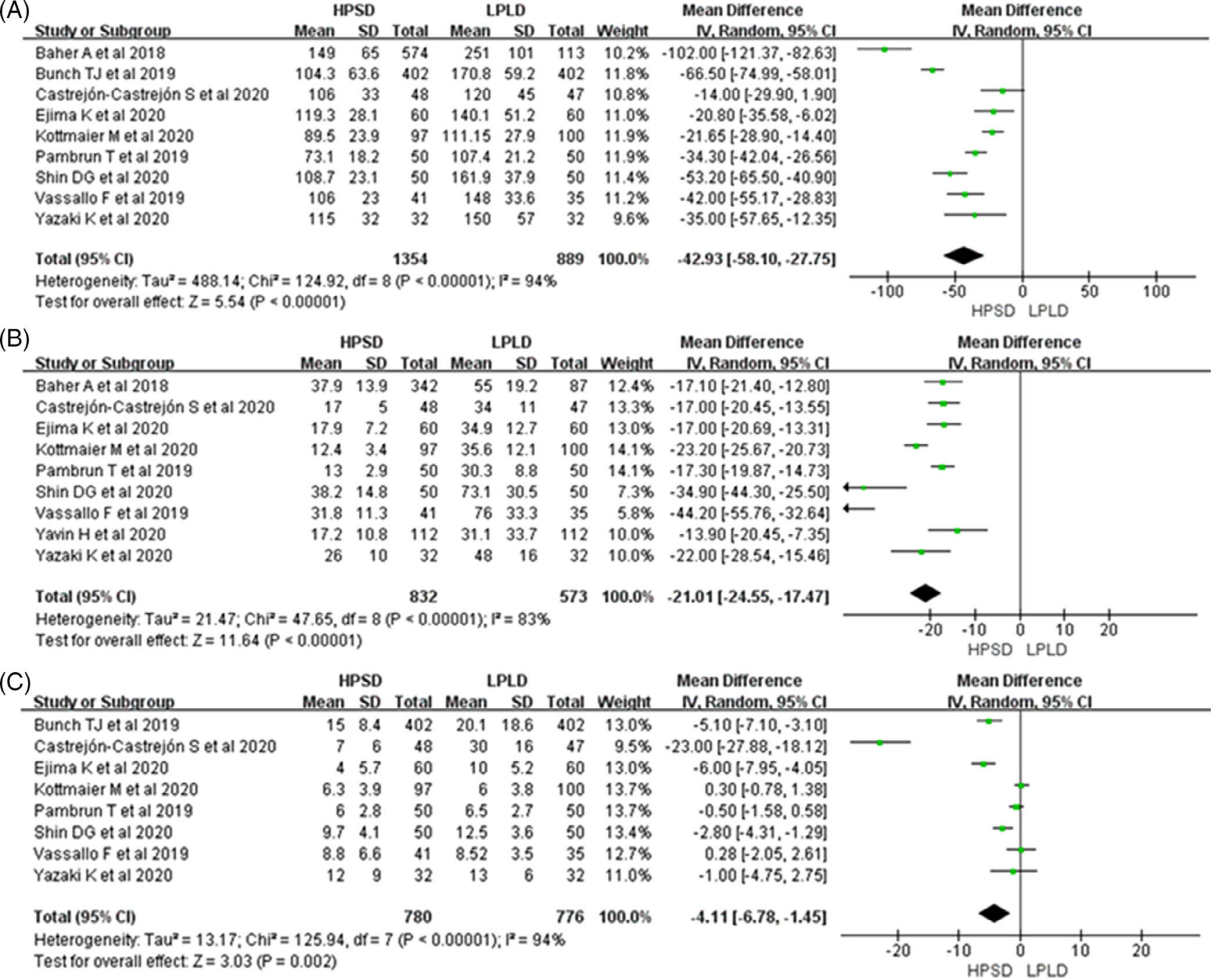
Forest plots of procedural time, A; ablation time, B; fluoroscopy time, C, for HPSD vs LPLD. HPSD, high power shorter duration; LPLD, low power longer duration

**FIGURE 4:**
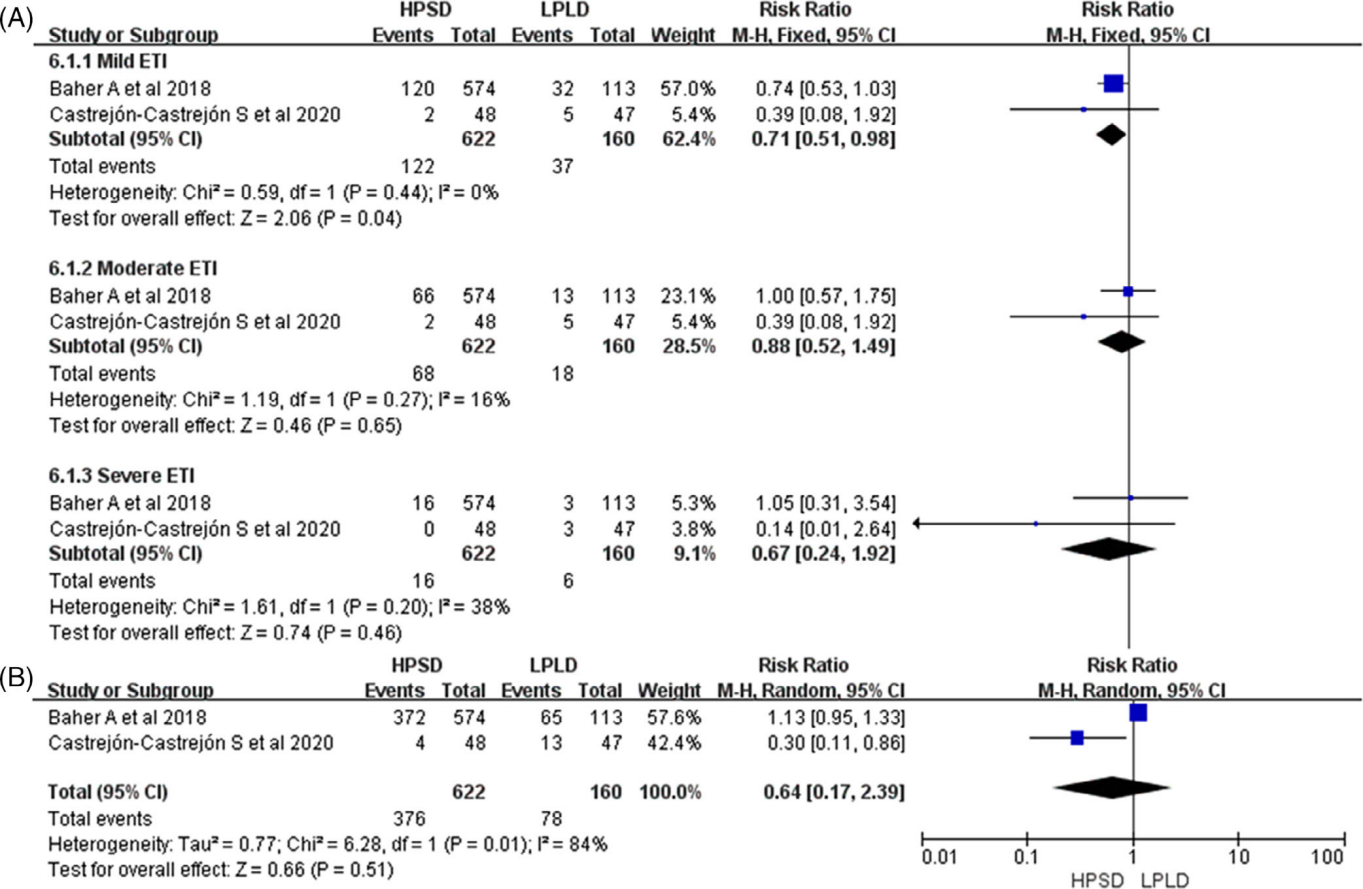
Forest plots of categories of ETI (A) and total ETI (B) for HPSD vs LPLD. ETI, esophageal thermal injury; HPSD, high power shorter duration; LPLD, low power longer duration

In all included studies, HPSD was found to be associated with a high rate of first‐pass PVI (RR: 1.20; 95% CI: 1.10‐1.31, *P* < .001 Figure 2A). Additionally, after an average follow‐up of 16 months, the pooled analysis indicated that HPSD could reduce the recurrence of atrial arrhythmias (RR: 0.73; 95% CI: 0.58‐0.91, *P* = .005; Figure 2B). Moreover, HPSD significantly reduced the total procedural times (WMD: −42.93; 95% CI, −58.10 to −27.75, *P* < .001; Figure 3A), ablation times (WMD: −21.01; 95% CI: −24.55 to −17.47, *P* < .001; Figure 3B), and fluoroscopy times (WMD: −4.11; 95% CI: −6.78 to −1.45, *P* < .001; Figure 3C). Major complications and ETIs were similar between two groups: (RR: 0.75; 95% CI: 0.44‐1.30, *P* = .31; Figure 2C) and (RR: 0.57; 95% CI: 0.21‐1.51, *P* = .51 Figure 4B); however, HPSD could reduce mild ETIs (RR: 0.64; 95% CI: 0.41‐0.99, *P* = .04 Figure 4A).

## 4 DISCUSSION

This study represented the first meta‐analysis that included the latest and high‐quality studies comparing HPSD and LPLD in patients with AF. The main findings were as follows: (a) HPSD was associated with higher first‐pass PVI and lower recurrence of atrial arrhythmias; (b) HPSD could significantly reduce procedural, ablation, and fluoroscopy times compared with LPLD; and (c) major complications and ETIs were similar between two groups.

HPSD, as a novel energy delivery strategy, was used to optimize LPLD. However, there are still some limitations with HPSD. This includes the ability of HPSD to create transmural lesions. Moreover, a high power does not denote an unlimited power increase. The upper limit of the power setting and how it affects the choice of ablation are still not known. These issues prompted us to conduct this meta‐analysis.

HPSD is largely based on immediate heating during the resistive phase. The average thickness of the left atrium is 2.8 ± 1.1 mm in humans.^25^ For patients with persistent AF, the mean atrial wall thickness is only 1.89 ± 0.5 mm and never exceeded 3.5 mm.^26^ HPSD affected tissues till a depth of 3.5 to 4 mm^2^; thus, the left atrial wall thickness is well within the depth for HPSD. Hence, HPSD is well suited for AF ablation. Several previous experiments had demonstrated the utility of HPSD. Bourier et al. found that HPSD created lesions similar in volume but wider and shallower than those of LPLD.^27^ In freshly killed porcine ventricles, Ali‐Ahmed et al. showed that for a given CF, 20s was needed to create a 4 mm‐deep lesion using a 20 W ablation, while only 6 to 7 seconds was sufficient for a 50 W ablation.^5^ Thus, HPSD could achieve a rapid and more controlled resistive tissue heating as well as avoid deeper collateral injuries. In clinical studies, Winkle et al. showed that HPSD using a CF sensing catheter, with a short procedure time and delivery of small amounts of total RF energy, was safe and resulted in excellent long‐term freedom from AF. No complications were reported in this trial.^28^

Ten studies were included in our analysis. During ablation, a CF sensing catheter and 3D electro‐anatomical mapping system were used. It is well known that the power and ablation times per site always change dynamically according to the left atrial anatomy. In the anterior of PVs, the power and ablation time would be increased appropriately, but in the posterior, the power and ablation time would be reduced. For the LPLD group, some studies performed ablation within a specific range (20‐40 W),^8^, ^17^, ^18^, ^19^, ^20^, ^23^, ^24^ and others had powers set to 30W.^16^, ^21^, ^22^ In our analysis, we defined LPLD as having an ablation power of 20 to 40 W for 10 to 30s per site. For the HPSD group, there was no consistent setting of power, according to published data. The power setting in most of the studies was 50 W,^8^, ^16^, ^18^, ^21^, ^24^ while three studies performed ablation with a setting of 45‐50 W,^20^, ^22^, ^23^ and one study used 70 W for ablation.^19^ The average ablation time per site was less than10s. Although there were differences in the power setting for the HPSD group, no significant impact on the bias and heterogeneity of the results was found. The likely reason may be that under stable CF, if a higher ablation power was applied, the ablation time would be shortened; otherwise, the ablation time would be appropriately extended by operators. The ablation powers and times can be varied accordingly within a certain range. Therefore, their injury index might be similar, which explained the homogeneity of the HPSD group. Moreover, to perform statistical analysis and explore the clinical benefits of HPSD more accurately, we defined HPSD as possessing a power of more than 40 W, with duration of 2 to 10s per site. Within the scope of our HPSD definition, these studies could be pooled for analysis.

This study showed a higher first‐pass PVI in the HPSD group than in the LPLD group. The main reasons may be the larger sizes, better uniformities, and better consistencies of the lesions created by HPSD than those by LPLD. Catheter‐tissue contact stability is an important factor contributing to lesion creation, and catheter instability in a constantly moving heart may account for the difficulty in transmitting heat to the tissue.^20^ The shortening of HPSD may mitigate the negative effects of catheter instability and likely optimize lesion creation by increasing the likelihood of keeping the catheter stable throughout the application.^28^ In the LPLD group, catheter stability was an issue when longer single‐lesion ablation was required, leading to unevenness of lesions, tissue edema, and a lower rate of first‐pass PVI.

HPSD may reduce the rate of long‐term recurrence of atrial arrhythmias. Complete PVI with transmural injury is most important for the freedom from AF during long‐term follow‐up.^29^, ^30^ During the initial ablation phase, the delivery of RF energy resulted in direct‐tissue heating during the resistive phase. This zone of direct heating served as a heat source for passive heat diffusion into deeper tissue layers during the conductive phase. As the ablation continued, lesion expansion occurred predominantly by convective heating. The acute lesion was composed of tissue heated to a lethal temperature and tissue heated to a sublethal temperature with reversible injury.^4^, ^18^ HPSD application increased the effect of resistive heating with high‐power and reduced conductive heating because of shorter energy delivery. Therefore, high‐power ablation may theoretically favor the creation of more transmural, continuous, and durable lesions. In the present study, HPSD reduced the recurrence of AF during the follow‐up period.

The pooled analysis showed significant advantages of procedural features in HPSD. The approach could shorten procedural and ablation time compared with the LPLD approach, thus limiting patient exposure to intravenous fluids and anesthesia. The fluoroscopy time was also shorter for the HPSD group, which had a direct favorable impact on the patient, operator, and supporting staff. These results were consistent with previously published findings.^5^, ^6^, ^15^, ^16^, ^22^, ^31^ A significant reduction in the procedural time was observed in the HPSD group because of the shorter ablation time when compared to that of LPLD. The shorter ablation time for HPSD was due to the shorter time required for lesion creation, higher first‐pass PVI, and fewer acute PV reconnections than those of LPLD.^2^ In the LPLD group, additional ablation was required for gap ablation in non‐transmural lesions and to achieve a biphasic block of PVs in order to obtain a complete PVI.^20^ During the procedure, catheter movement mostly relies on the combination of X‐ray and 3D electro‐anatomic mapping systems. Hence, longer ablation time in the LPLD group than in the HPSD group inevitably led to a greater fluoroscopy time to locate and move the catheter in the left atrium. Additionally, less irrigation fluid was needed during HPSD due to the shorter ablation time, making HPSD more suitable for patients with impaired left ventricular functions.

The level of safety related to using high power for AF ablation, especially on the posterior wall, was a concern.^32^ One study by Winkle et al. using HPSD ablation reported no increase in complications.^33^ According to the principle of HPSD, with increasing resistive and reducing conductive phases, minimizing damage to collateral tissues is a crucial consideration. Several animal studies suggested that HPSD was superior to LPLD with a lower complication rate than that with LPLD.^2^, ^4^ Some human studies using HPSD had shown excellent clinical outcomes with fewer complications when compared to those of LPLD.^14^, ^34^, ^35^ In the present study, the pooled analysis showed that the rate of complications was similar between the two groups. The AEF or ETI had rarely been reported because most studies were single‐center studies, and none were large enough to evaluate infrequent serious complications. A large observational study focused on the complications of the HPSD showed that: 13974 ablation procedures were performed on 10 284 patients, with an extremely low complication rate in the HPSD group. Only one AEF occurred in 11 436 ablation procedures using HPSD; however, three AEFs occurred in 2538 ablation procedures using LPLD.^31^ Two studies included in our analysis discussed ETI. The results showed low and similar rates of ETI, but HPSD could reduce mild ETI. No AEFs were reported in the included studies. Thus, HPSD was safe enough for AF ablation.^8^, ^17^ However, heterogeneity was observed in the pooled analysis of ETI because only two studies about ETI were included in our analysis; consequently, we could not use sensitivity analysis to identify the source of heterogeneity. We believe that more studies on ETI should be included in the future, and from those, we would obtain more homogeneous and reliable results.

AI is a novel ablation quality marker that incorporates stability, CF, time, and power in a weighted formula. Some studies have performed HPSD guided by AI^14^, ^15^ and have indicated significant clinical benefits. However, more clinical studies are needed to confirm this.

Another study reported by Reddy et al. compared LPLD with very high‐power‐short‐duration (vHPSD, delivery of 90 W for 4 seconds) using a QDOT microcatheter (Biosense Webster, Inc., CA, USA). They demonstrated that vHPSD was an efficient, feasible, and safe strategy for AF ablation. However, this study was not included in the present meta‐analysis due to a complete difference in settings of deliveries of vHPSD and HPSD. Nevertheless, vHPSD may be another promising strategy in the future.^6^

### 4.1 Study limitations

This study had some limitations. First, publication bias could not be completely excluded, and the inclusion of only published data contributed to that bias. Second, the number of included studies was limited to only 10, and most of the studies were designed as nonrandomized, except for one RCT. Therefore, more well‐designed and large‐scale RCTs are required to confirm these findings. Third, limited collateral tissue damage is one of the important advantages of HPSD; however, in the analysis, this damage was not completely reflected due to limited endpoints reported from the included studies, and the heterogeneity of ETIs shown by the pooled analysis. Fourth, the ablation power and duration settings in the included studies were not completely consistent.

## 5 CONCLUSIONS

This analysis demonstrated that HPSD was a feasible, efficient, safe, and effective approach for AF ablation. The approach had some significant advantages over LPLD, including reduced procedure, ablation, and fluoroscopy time. Simultaneously, HPSD was associated with a higher first‐pass PVI than that of LPLD and could reduce the recurrence of atrial arrhythmias than in LPLD cases. Moreover, HPSD was as safe as LPLD with a low rate of complications and ETI. However, there was no consensus about the power and duration settings for HPSD. Therefore, more clinical trials should be conducted to optimize dwell times, power settings, and even catheter selection to consistently create optimal lesions in the atrium.

## CONFLICT OF INTEREST

The authors declare no potential conflict of interest.

## AUTHOR CONTRIBUTIONS

Chao‐feng Chen and Jing Wu: contributed to write the manuscript; Chao‐feng Chen contributed to design of this work, statistical analysis, and wrote the article; Mei‐jun Liu: contributed to evaluate quality and retrieved the required data, Chao‐lun Jin: contributed to review the literature, performed the selection of the studies, and helped gather references for the manuscript. Dr Yi‐zhou Xu: contributed to design of this work, revised, and approved the final version of the manuscript.

## ETHICS STATEMENT

There were not any ethics problems in our paper.

## Supporting information


**Table S1** Characteristics of ablation settings

**Appendix** S1 Supporting Information

## Data Availability

The data that support the findings of this study are available from the corresponding author upon reasonable request.
