# Supplementary material for: Comparison of high‐power short‐duration and low‐power long‐duration radiofrequency ablation for treating atrial fibrillation: Systematic review and meta‐analysis
Source: Clin Cardiol. 2020 Oct 27;43(12):1631–40. doi: 10.1002/clc.23493 (PMC7724222; doi:10.1002/clc.23493)
Supplement: Supplementary file 1 — Table S1 Characteristics of ablation settings [file CLC-43-1631-s001.docx]

| Table 2 Characteristics of ablation settings. | | | | | |
| --- | --- | --- | --- | --- | --- |
| Trial (year) | Treatment group | Patients | Power  (W) | Ablation CF(g), ablation time per point(s) | Ablation  Strategy |
| Baher 2018 | HPSD | 574 | 50 | CF: 10‐20 g; Ablation time per point:5 s. | PVI+ line |
|  | LPLD | 113 | 25~35 | CF: 10‐20 g; Ablation time per point: 10~30 s. |  |
| Bunch 2019 | HPSD | 402 | 50 | CF: 10‐20 g; Ablation time per point: 2-10s. | PVI+ line |
|  | LPLD | 402 | 30 | CF: 5-20 g; Ablation time per point: 10-20s. |  |
| Castrejón-Castrejón 2020 | HPSD | 48 | 50~60 | CF: NR; Ablation time per point: 7±10 s. | PVI |
|  | LPLD | 47 | 20~30 | CF: NR; Ablation time per point: 30±60s. |  |
| Ejima K 2020 | HPSD | 60 | 50 | CF: 5‐20 g; Ablation time per point:9.1 ± 1.1 s. | PVI+ line |
|  | LPLD | 60 | 25~40 | CF: 10‐20 g; Ablation time per point: 25.0 ± 2.5 s. |  |
| Kottmaier 2020 | HPSD | 97 | 70 | CF: NR; Ablation time per point: 5 s. | PVI |
|  | LPLD | 100 | 30~40 | CF: NR; Ablation time per point:20±2.3 s. |  |
| Pambrun 2019 | HPSD | 50 | 40~50 | CF: 16.8±1.9 g; Ablation time per point:8.5±0.8 s. | PVI |
|  | LPLD | 50 | 25~30 | CF: 17.2±0.6 g; Ablation time per point:15.7±2.3 s. |  |
| Shin DG 2020 | HPSD | 50 | 50 | CF:12.1 ± 3.3 g; Ablation time per point: <10 s. | PVI+ line |
|  | LPLD | 50 | 30 | CF: 12.1 ± 4.0 g; Ablation time per point: <40 s. |  |
| Vassallo 2019 | HPSD | 41 | 45‐50 | CF: 8‐15/10‐20 g; Ablation time per point: 6 s. | PVI |
|  | LPLD | 35 | 30 | CF: 10‐30 g; Ablation time per point: 30 s. |  |
| Yavin H 2020 | HPSD | 60 | 45~50 | CF: 10.2 ±5.7 g; Ablation time per point 8s. | PVI |
|  | LPLD | 60 | 20~40 | CF: 10‐20 g; Ablation time per point 20 to 30 s. |  |
| Yazaki 2020 | HPSD | 32 | 50 | CF: 5‐15 g; Ablation time per point: 5–10 s. | PVI+ line |
|  | LPLD | 32 | 25~40 | CF: 10‐20 g; Ablation time per point: 30 s. |  |
| Values are reported as the mean ± SD, medians (interquartile range). HPSD: High power shorter duration, LPLD: Low power longer duration, PVI: Pulmonary vein isolation, CF: Contact force. NR: not recorded. | | | | | |
