# Supplementary figures and images for: Comparison of high‐power short‐duration and low‐power long‐duration radiofrequency ablation for treating atrial fibrillation: Systematic review and meta‐analysis
Source: Clin Cardiol. 2020 Oct 27;43(12):1631–40. doi: 10.1002/clc.23493 (PMC7724222; doi:10.1002/clc.23493)

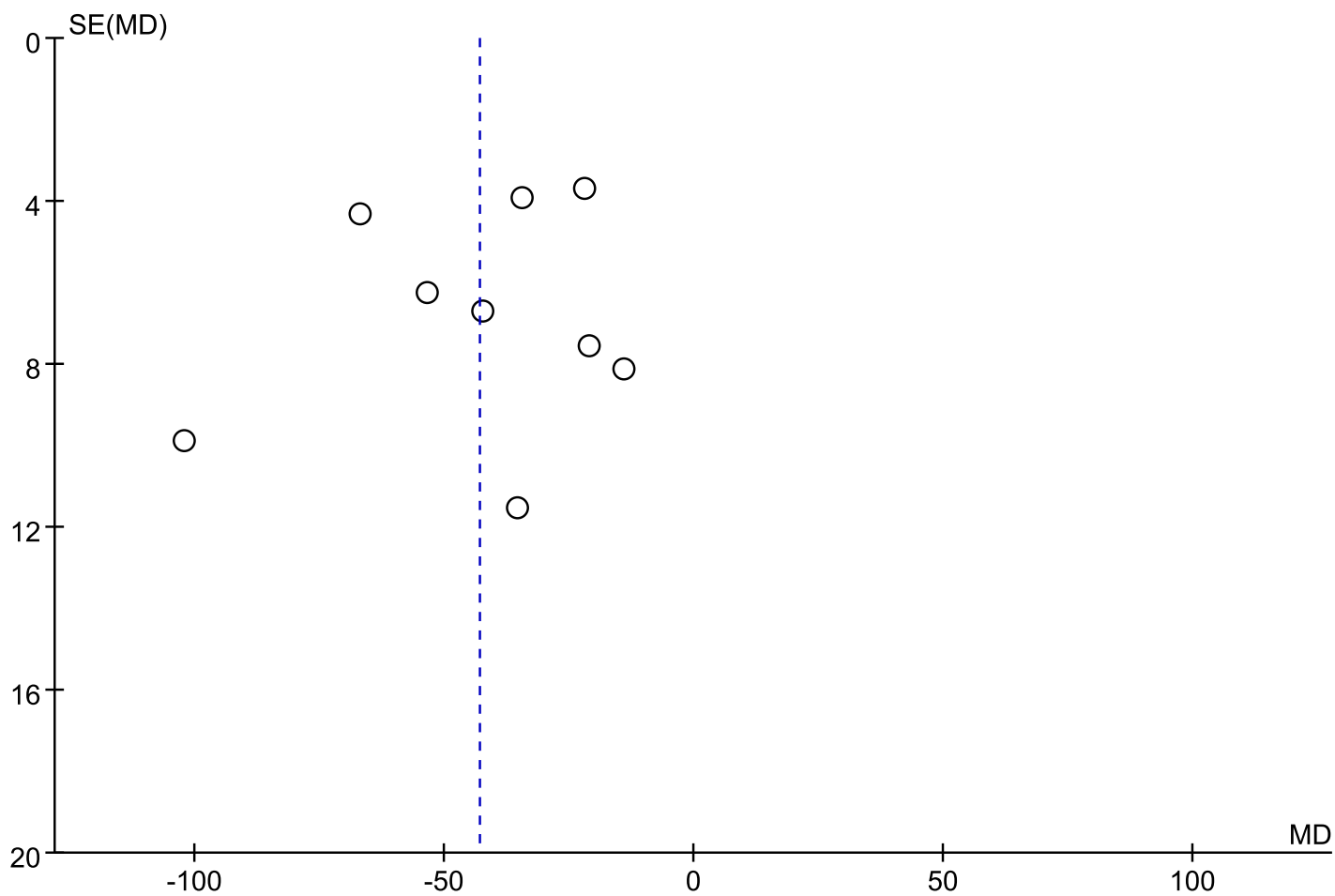

Supplement: Supplementary file 2 — Appendix S1 Supporting Information [file CLC-43-1631-s002.pdf]
